# Cognitive Assessment of Patients With Alzheimer's Disease by Telemedicine: Pilot Study

**DOI:** 10.2196/mental.8097

**Published:** 2018-05-11

**Authors:** Anna Carotenuto, Raffaele Rea, Enea Traini, Giovanna Ricci, Angiola Maria Fasanaro, Francesco Amenta

**Affiliations:** ^1^ Clinical Research, Telemedicine and Telepharmacy Centre School of Pharmacy University of Camerino Camerino Italy; ^2^ Bioethics and Legal Medicine Centre School of Law University of Camerino Camerino Italy; ^3^ Neurology Unit National Hospital (Antonio Cardarelli) Naples Italy

**Keywords:** dementia, telemedicine, videoconference, telepsychology, MMSE by videoconference, ADAS-cog by videoconference

## Abstract

**Background:**

Approximately 46.8 million people are living with dementia worldwide and their number will grow in the next years. Any potential treatment should be administered as early as possible because it is important to provide an early cognitive assessment and to regularly monitor the mental function of patients. Information and communication technologies can be helpful to reach and follow patients without displacing them, but there may be doubts about the reliability of cognitive tests performed by telemedicine.

**Objective:**

The purpose of this study was to evaluate the reliability of the Mini Mental State Examination (MMSE) and the Alzheimer’s Disease Assessment Scale cognitive subscale (ADAS-cog) tests administered in hospital by videoconference to patients with mild to moderate Alzheimer's disease.

**Methods:**

The tests were administered to 28 Alzheimer's disease outpatients (8 male, mean age 73.88, SD 7.45 years; 20 female mean age 76.00, SD 5.40 years) recruited and followed in the Alzheimer’s Unit of the A Cardarelli National Hospital (Naples, Italy) at baseline and after 6, 12, 18, and 24 months of observation. Patients were evaluated first face-to-face by a psychologist and then, after 2 weeks, by another psychologist via videoconference in hospital.

**Results:**

This study showed no differences in the MMSE and ADAS-cog scores when the tests were administered face-to-face or by videoconference, except in patients with more pronounced cognitive deficits (MMSE<17), in which the assessment via videoconference overestimated the cognitive impairment (face to face, MMSE mean 13.9, SD 4.9 and ADAS-cog mean 9.0, SD 3.8; videoconference, MMSE mean 42.8, SD 12.5 and ADAS-cog mean 56.9, SD 5.5).

**Conclusions:**

We found that videoconferencing is a reliable approach to document cognitive stability or decline, and to measure treatment effects in patients with mild to moderate dementia. A more extended study is needed to confirm these results.

## Introduction

Approximately 46.8 million people are living with dementia worldwide and it is expected that this figure will double every 20 years [1]. The number of people affected by dementia will probably reach 74.7 million in 2030 and 131.5 million in 2050 [2]. Alzheimer's disease (AD) is the most common type of dementia, representing between 60% and 80% of dementia cases [3]. The costs of this disease are very high now and will become even higher in the future, and will impact severely, directly, and indirectly on health systems and patients’ families [4]. No therapy has been found to stop dementia progression, but any potential treatment should be administered as early as possible. Therefore, it is crucial to provide a cognitive assessment of elderly patients as early as possible and continue monitoring it [5-7]. Information and communication technologies have been employed in dementia and they are particularly helpful [8], allowing physicians, psychologists, and nurses to reach patients without displacing them [9,10]. Several studies have evaluated the use of telemedicine for dementia disorders, but doubts still exist about the reliability of cognitive tests applied by videoconference [11,12].

In this field, telemedicine shows benefits and limitations. Among the benefits, there is the possibility to include patients who prefer to stay at home rather than going to the hospital or clinics [13] and for their caregivers to not be alone in providing better care. Costs and the displacement of patients can be reduced, more frequent monitoring can be assured, and waiting lists and hospital staff work are lowered and the caregiver time is preserved [14]. These benefits justify why cognitive assessment via videoconferencing is potentially useful and valid in dementia patients.

However, telemedicine also has some risks: older adults are frequently resistant to use new technologies and their use requires necessary skills. The security of sensitive data, the identification of the evaluator, and the quality of data transmission may represent further limitations [15].

The main concern is the reliability of data obtained by telemedicine. The assessment of AD requires the evaluation of cognitive functions, which is done through specific tests. These include the Mini Mental State Examination (MMSE), the most used for clinical purposes, and the Alzheimer’s Disease Assessment Scale cognitive subscale (ADAS-cog), the most used for measuring the effect of treatments. The feasibility of the MMSE by videoconference has been investigated by several authors [16-23] (Table 1). One of these studies was done in an Italian population. No differences were reported between the scores obtained when the MMSE was administered face-to-face versus videoconference, except in one study in which it was found for 40% of the patients the videoconference MMSE was two points lower than the face-to-face MMSE [21].

The majority of previous studies have considered mild to moderate AD outpatients, living in different contexts, more often in rural areas. All studies have used the traditional 30-item MMSE, except one which has used a 28-item MMSE [17]. In summary, MMSE studies in videoconference have not yet evaluated the follow-up of AD patients. No study, to our knowledge, has evaluated the reliability and feasibility of the ADAS-cog test by videoconference modality. Moreover, the feasibility of MMSE has not been fully evaluated. On this basis, we wanted to assess if MMSE and ADAS-cog are reliable at follow-up.

**Table 1:** Articles reviewed on “cognition screening tests by telemedicine” that used videoconferencing. AD: Alzheimer's disease; GDS: Global Deterioration Scale; MCI: mild cognitive impairment; MMSE: Mini Mental State Examination; VMMSE: Videoconference-based Mini Mental State Examination.

Authors	Year	Demographics of patients	Patients investigated	Results
Cullum et al [22]	2014	N=202; age: mean 68.5 (SD 9.5) years; education: mean 14.1 (SD 2.7) years	59% healthy controls and 41% with MCI or AD	VMMSE and face-to-face MMSE was comparable (with the score is >15)
Kim et al [16]	2017	N=188; age: mean 78 (SD 24) years; education: telemedicine (2.4 years) and face-to-face (3.4 years)	Mild-moderate dementia	The mean annual VMMSE changes were less than the mean face-to-face MMSE score changes (0.60 vs 1.03 points), but not statistically significant. More than 95% of participants were treated with cholinesterase inhibitors
Timpano et al [17]	2013	N=342 (134 male); age: range 50-94 years; education: range 0-18 years	Cognitively impaired and healthy patients	VMMSE is comparable with face-to-face MMSE, but with cut-off 28
Ciemins et al [18]	2009	N=63 (45% female); age: mean 61 years (range 36-90)	Type 2 diabetics, 17% with associated depression	≥95% concordance in the 80% of the items of VMMSE with face-to-face MMSE
Mc Eachern et al [20]	2008	N=71 (34 male); age: mean 72 (SD 11) years	37 AD, 11 MCI, 4 vascular dementia, 10 other pathology, 9 normal	No difference between VMMSE and face-to-face (*P*=.23)
Loh et al [19]	2007	N=20 (9 male); age: range 65-79 years	Cognitively impaired	The mean face-to-face MMSE was 23.3 (SD 3.6), MMSE by videoconference was 24.2 (SD 3.7)
Loh et al [21]	2004	N=20; age: mean 82 (range 72−95) years	Demented	VMMSE yielded similar results to face-to-face MMSE in 60% of patients; however, there was a moderate difference in 40% of two points or more on the MMSE on face-to-face MMSE
Montani et al [23]	1997	N=14; age: mean 88 (SD 5) years	Mixed	Mean scores VMMSE (22.2) were similar with face-to-face MMSE (23.7)

On this basis, our aims were (1) to evaluate if the videoconference administration of MMSE and ADAS-cog were comparable to the face-to-face administration, and (2) to assess the acceptance of patients and caregivers of the videoconference modality. Our study was focused on these aspects and not on the diagnosis of AD that in the majority of cases requires more extensive and articulated diagnostic procedures.

## Methods

### Participants

The study sample consisted of 28 AD outpatients (8 male, 20 female) followed by the Alzheimer and Neurodegenerative Diseases Unit, Neurology Department, A Cardarelli National Hospital in Naples, Italy. Supervision, organization, and informatics support and statistics were provided by the Clinical Research Centre of Camerino University in Camerino, Italy. Clinical diagnosis of AD was performed by a neurologist according to the National Institute of Neurological and Communicative Disorders and Stroke and the Alzheimer’s Disease and Related Disorders Association criteria. Moreover, brain magnetic resonance imaging was used to confirm the diagnosis. The severity of dementia was assessed by the MMSE, the Activities of Daily Living (ADL), the Instrumental Activities of Daily Living (IADL), the Clinical Dementia Rating, and other neuropsychological tests scores.

Inclusion criteria were age older than 50 years, MMSE score between 24 and 12, education for more than 5 years, good visual-hearing ability, and living with or in contact with a caregiver willing to cooperate in the evaluation of effectiveness. All patients were community dwelling and were enrolled in the study at least 6 months after the diagnosis. Exclusion criteria were decompensated heart disease, chronic renal failure, severe liver failure, uncorrected dysthyroidism, cancer; diagnosis of major depression (according to *DSM-IV* criteria), and a different diagnosis of AD. Patients were randomly recruited among outpatients followed by the Alzheimer and Neurodegenerative Diseases Unit.

The mean age of male patients was 73.88 (SD 7.45) years and of female patients was 76.00 (SD 5.40) years. All 28 patients had a mean education of 7.61 (SD 4.07) years. Sixteen patients were widowed and 12 were married. All were retired and lived at home, with 12 patients living with their spouse, 11 with a child, and 5 with non-family caregivers. Six patients were left-handed. Comorbid health conditions were high blood pressure (n=27), hypercholesterolemia (n=26), diabetes (n=4), and ischemic cardiopathy (n=6). No patients had comorbidities of psychiatric disorders, although 14 patients had anxiety. All patients and their caregivers signed an informed consent form. A questionnaire developed specifically for this study was used to assess the level of acceptance of the telehealth procedures. This study was reviewed and approved by the ethical committee of A Carderelli Hospital.

### Tests and Questionnaire

The Italian versions of the MMSE [24] and ADAS-cog [25] were used for each participant in both the face-to-face and videoconference modalities.

The MMSE is a brief, quantitative measure of the cognitive status of adults. It can be used to screen for cognitive impairment, to estimate the severity of cognitive impairment at a given point in time, to follow the course of cognitive changes in an individual over time, and to document a patient’s response to treatment. It consists of 30 items (questions), which refer to seven different cognitive areas: time orientation, spatial orientation, registration, attention and calculation, recall, language, and praxis. The total score ranges from a minimum of zero and a maximum of 30 points [26]. The normative study in an Italian elderly population found that a score of 24 or less, corrected by age and educational level using the score-adjustment coefficients, is suggestive of dementia; lower scores indicate greater cognitive impairment [24]. Administration time is approximately 10 to 15 minutes. A meta-analysis showed that the MMSE has a sensitivity of 79.8%, a specificity of 81.3%, a positive predictive value of 86.3%, and a negative predictive value of 73.0% for dementia [27]. For conversion from mild cognitive impairment (MCI) to AD, the accuracy of MMSE scores ranged from sensitivities of 27% to 89% and specificities from 32% to 90% [28].

The ADAS-cog measures the cognitive performance of six broad areas of cognition: memory; language; ability to orient oneself to time, place, and person; construction of simple designs and planning; and performing simple behaviors in pursuit of a basic, predefined goal [29]. The ADAS-cog is scored from zero to 70; higher scores indicate greater cognitive impairment. Administration time is approximately 30 to 45 minutes. The Italian study of ADAS-cog on psychometric and normative data was based on a sample of 95 healthy volunteers. Results indicated a specific influence of patient educational level on the cognitive subscale total score of ADAS and the need for an adequate correction was observed [25]. The ADAS-cog cut-off score for dementia was 12 or less with sensitivity and specificity values of 89.19% and 88.53%, respectively [30]. The best cut-off score of ADAS-cog to distinguish between MCI and AD was 12 or higher with sensitivity of 0.86, specificity of 0.89, positive predictive values of 0.99, and negative predictive values of 0.32 [31].

A questionnaire on the acceptance of the videoconference modality for cognitive testing included five questions with a response ranging from 1 to 5, where 1=I strongly disagree and 5=I strongly agree. This questionnaire assessed the experience of videoconferencing, including an overall evaluation, if participants wanted to repeat the experience, attitudes toward data privacy, and clarity of instructions. The questionnaire was administered to both the patient and the caregiver.

### Materials and Videoconferencing System Utilized in Hospital

Two Sony VAIO laptops were used for videoconferencing and data collection. The Sony VAIO laptops contained an IntelCore Duo CPU P8400 2.26 GHz processor, 4 GB memory, Intel Media Accelerator X3100 graphics card, and a 17.3″ LCD LED (1920×1080) integrated screen. The videoconferencing system used was the BCC950 Logitech, with integrated microphone and video camera. Computers operated under the corporate domain, restriction of use policies, and antivirus systems.

Administration of the MMSE and ADAS-cog tests took place through real-time videoconferencing, on both terminals, with Microsoft Skype. Connection speed of the local area network of Cardarelli Hospital connection averaged 100 Mbit/s and had perimeter firewalls to guarantee security protection of the connection.

To evaluate the reliably of the videoconference, both the psychologist’s and patient’s computers were connected to the same LAN. Videoconference ensured high levels of security and encrypted communication. Patients were prepared in advance for the possibility of occasional technological problems, such as when calls “drop out” or the video image becomes frozen. Remote control of the audiovisual system was done with Virtual Network Computing software.

### Procedure

After obtaining informed consent by a research assistant, the MMSE and ADAS-cog tests were administered by face-to-face assessment and videoconference modalities. Both the face-to-face and videoconference assessments were done in the hospital. Tests were administered at baseline and after 6, 12, 18, and 24 months. All patients had been previously diagnosed in the Alzheimer Unit of Cardarelli Hospital by a neurologist. The screening for eligibility criteria was made by the neurologist. Patients were evaluated first face-to-face by a psychologist for inclusion criteria (MMSE score between 24 and 12). For each patient, tests were administered five times (baseline and after 6, 12, 18, and 24 months) in both face-to-face and videoconference modalities. The interval between each administration was 2 weeks to minimize any practice effect. The administration was done by two blinded psychologists independently (rater 1 and rater 2).

In the task of “naming objects,” real objects were used, whereas the ‘‘close your eyes” command was presented in full screen and shown to the patient via the webcam as well as the pentagon drawing task. Psychologists were trained in administering the MMSE and ADAS-cog by face-to-face and by videoconference, and had more than 10 years’ of experience. Caregivers were not present during the cognitive assessments, but an assistant was present in case of technical issues.

The videoconference modalities took place in a hospital room equipped with the instruments and an informatics operator participated to check if the visual and audio settings were adequate. The time required in both conditions (face-to-face and videoconference) was also measured.

### Statistics

The ANOVA test was used to assess if the administration modality (independent variable) was associated with any difference in total scores of MMSE and ADAS-cog tests (dependent variables). A further analysis considered the subgroups of patients according to their MMSE at baseline. For this analysis, they were grouped by MMSE score as slightly impaired (score 21-24), moderately impaired (score 18-20), and severely impaired (score 15-17). The ANOVA test was used for assessing the significance of differences between the preceding patient groups.

## Results

A total of 28 AD patients (8 male, 20 female) with a mean baseline MMSE of 19.6 (SD 3.0), ADL mean 3.1 (SD 1.0), and IADL mean 2.0 (SD 0.8) were evaluated by administering the MMSE and ADAS-cog in both a face-to-face and videoconference modality. All participants reached the end of the study period of 2 years.

Baseline and follow-up MMSE scores obtained by face-to-face or videoconference modality are summarized in Table 2. As shown, no significant differences were noticeable between face-to-face or videoconference testings. The same was true for baseline and follow-up ADAS-cog scores, shown in Table 2. The time required for performing the tests was also comparable, with mean 37 (SD 8) minutes in the face-to-face modality and mean 38 (SD 10) minutes in the videoconferencing modality.

Data derived from the MMSE and ADAS-cog tests were also analyzed separately, dividing patients into three groups according to their MMSE scores at baseline recorded at the enrollment (eg, severely impaired, moderately impaired, and slightly impaired). As shown in Table 3, at baseline and follow-up, the two modalities did not influence the MMSE scores of the first and second groups of patients. Patients in the third group, who had a lower MMSE, obtained lower scores by the videoconference modality compared to the face-to-face modality. The same differences were observed in the ADAS-cog test (Table 4) with patients in the third group having higher scores by videoconference than in the face-to-face modality.

**Table 2:** The Mini Mental State Examination (MMSE) and Alzheimer’s Disease Assessment Scale cognitive subscale (ADAS-cog) values obtained by face-to-face or videoconference modalities.

Tests		Baseline	6 months	12 months	18 months	24 months
**MMSE**						
	Face-to-face, mean (SD)	19.6 (3.0)	19.5 (5.0)	18.4 (5.8)	18.3 (6.1)	17.8 (6.8)
	Videoconference, mean (SD)	18.8 (4.5)	18.7 (5.4)	17.7 (6.5)	17.3 (7.1)	16.3 (7.7)
	*P* value	.37	.56	.68	.61	.42
**ADAS-cog**						
	Face-to-face, mean (SD)	28.6 (19.3)	29.3 (19.8)	31.9 (20.3)	33.9 (20.7)	34.8 (20.0)
	Videoconference, mean (SD)	34.1 (17.4)	34.5 (17.4)	36.5 (17.4)	39.7 (15.1)	40.4 (13.5)
	*P* value	.07	.07	.19	.12	.17

**Table 3:** The Mini Mental State Examination (MMSE) values obtained by face-to-face or videoconference in patients with baseline MMSE showing slight (21-24), moderate (18-20), or severe (15-17) impairment.

MMSE impairment level		Baseline	6 months	12 months	18 months	24 months
**Slight**						
	Face-to-face, mean (SD)	23.0 (1.1)	24.5 (3.2)	24.6 (2.7)	25.4 (2.5)	24.7 (3.3)
	Videoconference, mean (SD)	23.1 (1.5)	24.30 (2.3)	24.5 (2.3)	25.6 (2.5)	24.7 (2.9)
	*P* value	.87	.88	.93	.93	>.99
**Moderate**						
	Face-to-face, mean (SD)	19.3 (0.9)	18.0 (2.2)	16.4 (2.9)	16.1 (2.9)	14.1 (5.1)
	Videoconference, mean (SD)	19.0 (1.2)	18.3 (2.1)	17.1 (2.8)	16.2 (3.1)	14.1 (4.9)
	*P* value	.51	.75	.63	.94	>.99
**Severe**						
	Face-to-face, mean (SD)	16.1 (0.8)	15.4 (3.9)	13.3 (3.9)	13.2 (3.9)	13.9 (4.9)
	Videoconference, mean (SD)	12.7 (1.5)	12.8 (2.6)	10.7 (3.8)	10.2 (3.7)	9.0 (3.8)
	*P* value	<.001	.11	.17	.11	.03

**Table 4:** The Alzheimer’s Disease Assessment Scale cognitive subscale (ADAS-cog) values obtained by face-to-face or videoconference in patients with baseline Mini Mental State Examination (MMSE) showing slight (21-24), moderate (18-20), or severe (15-17) impairment.

MMSE impairment level		Baseline	6 months	12 months	18 months	24 months
**Slight**						
	Face-to-face, mean (SD)	22.5 (5.7)	21.8 (3.8)	22.5 (9.7)	23.8 (11.1)	24.0 (11.4)
	Videoconference, mean (SD)	23.0 (5.3)	22.0 (3.5)	22.8 (6.8)	26.0 (12.8)	24.5 (11.6)
	*P* value	.84	.90	.94	.70	.92
**Moderate**						
	Face-to-face, mean (SD)	29.2 (4.4)	31.8 (4.8)	34.0 (5.7)	35.6 (7.2)	38.8 (10.5)
	Videoconference, mean (SD)	30.2 (4.9)	33.0 (4.1)	34.9 (5.1)	38.7 (6.7)	41.4 (9.5)
	*P* value	.65	.57	.73	.36	.58
**Severe**						
	Face-to-face, mean (SD)	34.9 (7.0)	35.1 (7.3)	40.3 (9.5)	42.4 (10.3)	42.8 (12.5)
	Videoconference, mean (SD)	52.4 (7.6)	49.9 (6.9)	53.2 (7.7)	54.6 (6.7)	56.9 (5.5)
	*P* value	<.001	<.001	.01	.01	.01

**Table 5:** Results of the questionnaire^a^ on acceptance of the videoconference modality for cognitive testing.

Questions	Patients, mean (SD)	Caregivers, mean (SD)
1. Instructions are clear and understandable	4.4 (1.3)	4.6 (0.9)
2. Data privacy is assured	4.8 (0.6)	4.5 (1.1)
3. I saved my time	4.0 (0.8)	4.8 (0.5)
4. I would like to repeat the experience	4.5 (0.8)	4.3 (1.3)
5. I prefer the Web modality than coming to the hospital	3.3 (1.5)	4.3 (1.5)

^a^Significance of questionnaire’s ranking: 5=strongly agree; 4=agree; 3=neutral; 2=disagree; 1=strongly disagree.

The acceptance of videoconference examination documented by a short questionnaire was quite high, with both patients and caregivers appreciating this modality. In particular, the preference for the web modality versus coming to the hospital was very high for both patients (mean 3.3, SD 1.5) and caregivers (mean 4.3, SD 1.5; Table 5).

## Discussion

In this study, we evaluated the reliability and feasibility of the MMSE and ADAS-cog tests by videoconference in mild to moderate AD outpatients. Two blinded raters administered the tests by face-to-face and videoconference modalities and compared the results. The MMSE results were the same in the two modalities. Only in nine patients with more pronounced cognitive deficits (MMSE<17) did the videoconference modality overestimate the impairment.

The results from the MMSE are consistent with findings reported by other studies [16-23]. Most studies evaluated patients affected by dementia or MCI, except in one study [18] that examined the reliability of MMSE administration via remote administration in a group of 72 patients with diabetes. These studies did not find differences between the scores of the MMSE administered face-to-face versus videoconference or telephone interviews [16-23]. In contrast to a larger previous study by another group [17], we used the traditional 30-item MMSE, which includes the writing and drawing tasks.

For the ADAS-cog test, this is the first study, to our knowledge, using a videoconference modality. In our investigation, we observed that the face-to-face or videoconference modalities did not influence the ADAS-cog scores of the first and second group of patients (MMSE scores 21-24 and 18-20, respectively), whereas patients in the third group, who had a lower MMSE (15-17), obtained more severe (higher) scores by videoconference compared to the face-to-face modality. This is probably due to the difficulty of understanding the meaning of specific questions. These findings suggest using the videoconference modality with patients with mild to moderate AD and excluding those with a moderate to severe AD.

Videoconferencing has been shown to be reliable not only in AD, but to assess the cognitive functions in other pathologies. Interesting results were provided for psychiatric patients [32-33], patients with Parkinson disease [34], and stroke patients [35], as well as in older adults [36]. Furthermore, videoconferencing has been found useful to evaluate the clinical status [37] and rehabilitation [38] of patients affected by different neurological disorders. All the preceding studies confirm that videoconference instructions are clear enough. This also was observed in our sample of patients, who reported that for saving time they preferred the teleconference modality than coming to the hospital. That is one good reason for supporting the videoconference approach. The patients and their caregivers were informed that videoconference evaluation ensured high levels of security and encrypted communication. From responses to the questionnaire on acceptance, they did not have concerns about the privacy of their medical information. The acceptability of the videoconference modality in this study is consistent with previous investigations in AD, which reported that 98% of the patients were satisfied and felt at ease with it [32,39].

Previous studies found that women, older patients, and less educated patients may be less receptive to technology. In contrast, men, younger patients, and those with higher education are more receptive to telemedicine and report less anxiety [40,41]. The link found between education and technology acceptability is worth being considered. Studies have revealed that higher levels of education are associated with higher levels of both computer knowledge and computer interest and lower levels of computer anxiety [42].

The range of settings where videoconferencing can be used is wide and it can represent a useful and effective method for assessing cognitive functions. However, continued validation studies and adaptation of neuropsychological instruments is warranted.

Ethical and legal consequences of assessing cognitive functions via telemedicine deserve to be discussed as well. Caring for patients suffering from dementia poses complex ethical problems because of the nature of dementia and the way that dominant ethical principles apply to its clinical features [43]. These problems include informed consent, the duty to maintain the confidentiality, and privacy of patient examination and records [44-48]. In this study, patients and/or their caregivers were informed about potentiality and the limits of the videoconference approach used and in our sample and none reported any issues regarding privacy. However, the study was performed in a dedicated hospital setting where patients were routinely examined. The option of carrying out remote videoconferences with patients staying at home was not applied; therefore, all ethical implications of home-based cognitive function assessment should be further investigated.

Our study has strengths and limitations. The long duration (24 months) of observation, the well-defined diagnosis, and comparable clinical characteristics of all patients, which make the sample quite homogenous, are strengths of our study. In fact, only sparse studies have evaluated MMSE and ADAS-cog by videoconference for follow-up. The presence of two independent and blinded raters with specific experiences should be also positively considered.

On the other side, we recognize that the number of patients investigated is obviously small because this was a pilot study. We are also aware that we observed patients in a hospital setting with the presence of an assistant. This was done to check the audiovisual system. However, we cannot exclude that the same patients could behave differently if they were at home. These limits should be addressed in a future study with a larger sample group.

Despite these possible limitations, the MMSE and ADAS-cog administration via telemedicine is useful to simplify the assessment of patients and to allow wider participation to clinical trials of people living in remote geographical areas. Further research with a larger sample group and a remote geographical location are required.

In conclusion, videoconferencing can be used to assess patients with mild to moderate dementia, document their cognitive stability or decline, and measure the effects of the treatments.

## Abbreviations

AD: Alzheimer's disease
ADAS-cog: Alzheimer’s Disease Assessment Scale cognitive subscale
ADL: Activities of Daily Living
IADL: Instrumental Activities of Daily Living
MCI: mild cognitive impairment
MMSE: Mini Mental State Examination
